# Electrical Impedance Tomography–Guided Airway Clearance in Elderly Patients With Severe Pneumonia: A Prospective Study

**DOI:** 10.1111/crj.70110

**Published:** 2025-07-13

**Authors:** Jiaping Zhao, Wenchao Mao, Yi Zhang, Saichan Xu, Fei Qian, Liang Wu, Shijin Gong, Weihang Hu, Changyun Zhao

**Affiliations:** ^1^ Department of Critical Care Medicine Zhejiang Hospital Hangzhou Zhejiang China; ^2^ Department of Critical Care Medicine Quzhou Kecheng People's Hospital Quzhou Zhejiang China

**Keywords:** airway clearance, elderly, electrical impedance tomography, respiratory mechanics, severe pneumonia

## Abstract

**Background:**

Elderly patients are prone to secretion retention and exacerbated lung infections due to weakened respiratory muscle strength and reduced ability to cough and expectorate. Airway clearance techniques (ACTs) can help to clear airway secretions, but objective bedside assessment of secretion clearance efficacy is lacking. Electrical impedance tomography (EIT) can dynamically monitor lung ventilation and provide a basis for clinical decision‐making.

**Methods:**

This study was a prospective randomized controlled trial that included 50 elderly patients with severe pneumonia, who were randomized into EIT and non‐EIT groups. The EIT group received personalized ACTs guided by real‐time EIT imaging with dynamic adjustment of posture, percussion intensity, and active circulatory breathing technique (ACBT) frequency, whereas the non‐EIT group received fixed‐schedule ACTs (postural drainage every 2 h + percussion/vibration twice daily) without EIT feedback. The main observation indices included Clinical Pulmonary Infection Score (CPIS), respiratory mechanics indices, blood gas analysis indices, and extubation success rate.

**Results:**

The EIT group showed significantly lower CPIS scores (*p* = 0.0137 on Day 7), higher dynamic compliance (*p* = 0.0193), lower airway resistance (*p* = 0.0039), lower peak airway pressure (*p* = 0.0288), and higher oxygenation index (*p* = 0.0143 on Day 5 and *p* = 0.0005 on Day 7) than the non‐EIT group. The extubation success rate was significantly higher in the EIT group (88% vs. 56%, *p* = 0.0255). Additionally, the EIT group demonstrated progressive improvements in ventilation in specific regions (D7 vs. D1: *p* = 0.0004 for region of interest [ROI]3; *p* = 0.0059 for ROI4) and a significant decrease in the global inhomogeneity index at D7 (D7 vs. D1: *p* = 0.0025).

**Conclusion:**

EIT‐guided ACT is safe and enhances treatment efficacy by significantly improving respiratory function and extubation success rate in elderly patients with severe pneumonia.

## Graphical abstract

EIT‐guided personalized airway clearance significantly improves outcomes in elderly severe pneumonia patients. Real‐time electrical impedance tomography (EIT) optimized secretion clearance through dynamic adjustment of posture, percussion, and breathing techniques. This approach enhanced respiratory mechanics, oxygenation, and infection control, doubling extubation success rates (88% vs. 56%) compared with standard fixed‐schedule therapy.

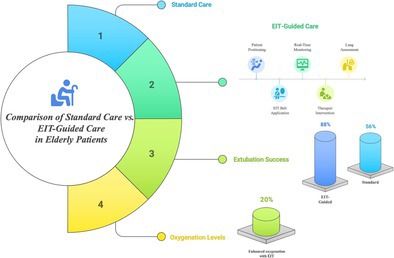

## 1 Introduction

Pneumonia is one of the leading causes of infectious morbidity and mortality in elderly patients [1]. Among elderly individuals with pneumonia, a pronounced attenuation of respiratory muscle strength culminates in a marked reduction of both the power and stamina of these muscles [2]. This impairment hampers the capacity for effective coughing and expectoration, leading to impaired sputum drainage and ultimately aggravating the infectious process [3]. Airway clearance techniques (ACTs) refer to a range of methods designed to enhance the removal of secretions from the airways, thereby promoting clear and unobstructed airflow [4]. Given the wide variety of assessment and operative techniques [5], there is an urgent need for accurate assessment and judgment to select the most appropriate technique and standardize its application according to the specific patient situation in clinical practice. In this context, electrical impedance tomography (EIT), as an emerging noninvasive and nonradioactive bedside monitoring method, allows clinicians to dynamically assess the regional ventilatory status of the lungs and provide important information for clinical decision making [6].

The EIT technique evaluates respiratory status and regional lung ventilation by measuring voltage changes on the body surface and analyzing thoracic conductivity distribution [7]. Its unique advantage lies in its ability to assess in real time the distribution of ventilation in the lung region and changes in end‐expiratory pulmonary impedance, providing visual, timely, and convenient monitoring for the adjustment of ventilator parameters and the achievement of pulmonary re‐expansion in patients with Acute Respiratory Distress Syndrome (ARDS) [8, 9]. This real‐time monitoring capability is expected to make EIT an ideal tool for guiding patients in the selection and adjustment of ACTs, as well as for continuous monitoring and assessment of pulmonary ventilation status during therapy.

The aim of this study was to evaluate the clinical efficacy of combining ACTs with EIT in mechanically ventilated elderly patients with severe pneumonia through a prospective randomized controlled trial approach. We hypothesized that EIT‐guided personalization of ACTs would improve therapeutic efficacy and prognosis.

## 2 Materials and Methods

### 2.1 Patients

This prospective, observational study followed the tenets of the Declaration of Helsinki and was conducted in two mixed Intensive Care Units (ICUs) of tertiary hospitals in China over a period of 22 months. The protocol was approved by the ethics committee of Zhejiang Hospital (approval number 2020‐110K). Written informed consent was obtained from each patient's next of kin before participation. All patient information was deidentified to ensure patient confidentiality and to prevent any form of identification. The reporting of this study conforms to the STROBE guidelines [10].

Patients were eligible if they fulfilled the following inclusion criteria: (1) met diagnostic criteria for severe pneumonia; (2) had mechanical ventilation duration of ≥ 7 days; and (3) were aged ≥ 60 years. The exclusion criteria were as follows: (1) patients with severe hemodynamic instability; (2) cases of moderate‐to‐large pleural effusion or pneumothorax without prior intervention; (3) patients with temporary or permanent cardiac pacemaker implantation; (4) individuals with spinal pathologies or specific postural requirements; (5) subjects requiring renal replacement therapy or extracorporeal membrane oxygenation (ECMO) support; (6) patients requiring mechanical ventilation for other clinical indications; and (7) patient's next of kin refused participation. Diagnostic criteria for severe pneumonia are as follows [11]: Diagnosis is established if either one major criterion or ≥ three minor criteria are met. The major criteria are (1) requirement for mechanical ventilation and (2) septic shock requiring vasopressor therapy. The minor criteria are (1) respiratory rate of ≥ 30 breaths/min; (2) PaO_2_/FiO_2_ ratio of ≤ 250 mmHg; (3) multilobar infiltrates on chest imaging; (4) confusion/disorientation; (5) blood urea nitrogen (BUN) of ≥ 20 mg/dL; (6) leukopenia (white blood cell [WBC] < 4 × 10^9^/L); (7) thrombocytopenia (platelet count < 100 × 10^9^/L); (8) hypothermia (core temperature < 36°C); and (9) hypotension requiring aggressive fluid resuscitation.

### 2.2 Treatment Methods

Participants were prospectively randomized into two groups: The non‐EIT group received standard respiratory therapy, and the EIT group received standard therapy supplemented by EIT‐guided interventions. In the non‐EIT group, airway management consisted of postural adjustment (elevation of the bed by 30°–40° after exclusion of contraindications), secretion control, oral hygiene, active airway humidification/warming, and tracheal cuff pressure monitoring (target value of approximately 25 cm H_2_O every 6 h). ACTs included chest percussion/vibration twice daily, postural drainage every 2 h [12], and active circulatory breathing technique (ACBT) twice daily [13]. The EIT group maintained the same airway management but used EIT‐guided clearance techniques based on real‐time ventilation imaging. EIT‐derived parameters guided the selection and intensity of ACTs. Postural drainage positions (e.g., lateral decubitus or prone) were adjusted to enhance ventilation in poorly aerated regions of interest (ROIs 3–4) as identified by EIT. Percussion and vibration were intensified if the combined ventilation of ROIs 3–4 fell below 35% of the total tidal variation. The frequency of ACBT cycles was increased from twice daily to every 4 h if the Global Inhomogeneity Index (GI) remained above 0.5 following initial clearance efforts. Additionally, the duration of each therapy session was extended by 50% if the tidal impedance in the dorsal regions (ROIs 3–4) failed to increase by at least 10% after standard intervention. After each intervention, the efficacy of airway clearance was immediately reassessed using real‐time EIT. These adjustments were repeated until the GI decreased to below 0.4 or the combined ventilation of ROIs 3–4 exceeded 40% of the total tidal variation.

EIT was performed using a PulmoVista 500 system (Dräger Medical) with 16 electrode strips located at the 4th/5th intercostal and abdominal reference electrodes. Ventilation distribution was analyzed by means of four isometric ROIs: ROIs 1–2 (ventral/nondependent) and ROIs 3–4 (dorsal/dependent), and tidal ratios were dynamically displayed on a monitor to optimize clearance goals [14].

### 2.3 Measurements and Data Collection

The following parameters were recorded: demographic data; laboratory indicators: comorbidities, Acute Physiology and Chronic Health Evaluation (APACHE) II score, and Clinical Pulmonary Infection Score (CPIS); respiratory mechanics indicators: dynamic compliance (Cdyn), peak airway pressure (Ppeak), and airway resistance (Raw); blood gas analysis indicators: oxygenation index (OI); and gas distribution indicators: ROI1, ROI2, ROI3, ROI4 regional ventilation ratio, and GI. GI was determined at each individual time point based on the regional ventilation data obtained from the four regions of interest (ROIs: 1–2 ventral/nondependent and 3–4 dorsal/dependent), according to the following standardized formula [15]:

$$\mathrm{GI}=\frac{\sum_{\text{pixellung}}\left[\Delta Z_{\text{pixel}}-\text{Median}\left(\Delta Z_{\text{lung}}\right)\right]}{\sum_{\text{pixellung}}\Delta Z_{\text{pixel}}}$$

### 2.4 Statistical Analysis

The results are expressed as the median (interquartile range) for quantitative variables and the number and percentage for categorical variables. An independent group *t*‐test was applied for normally distributed variables, whereas the Mann–Whitney U test was used for nonnormally distributed variables. Categorical variables were analyzed by the Chi‐square test or Fisher's exact test. Differences between time periods for the same indicator were analyzed using repeated measures ANOVA. Multivariable logistic regression was employed to assess the success rate of weaning for both groups, adjusting for potential confounders such as age, APACHE II score, and gender. A two‐tailed *p*‐value ≤ 0.05 was considered statistically significant. All data were analyzed using R version 4.0.2.

## 3 Results

### 3.1 Demographic and Clinical Characteristics of the EIT and Non‐EIT Groups

**FIGURE 1:**
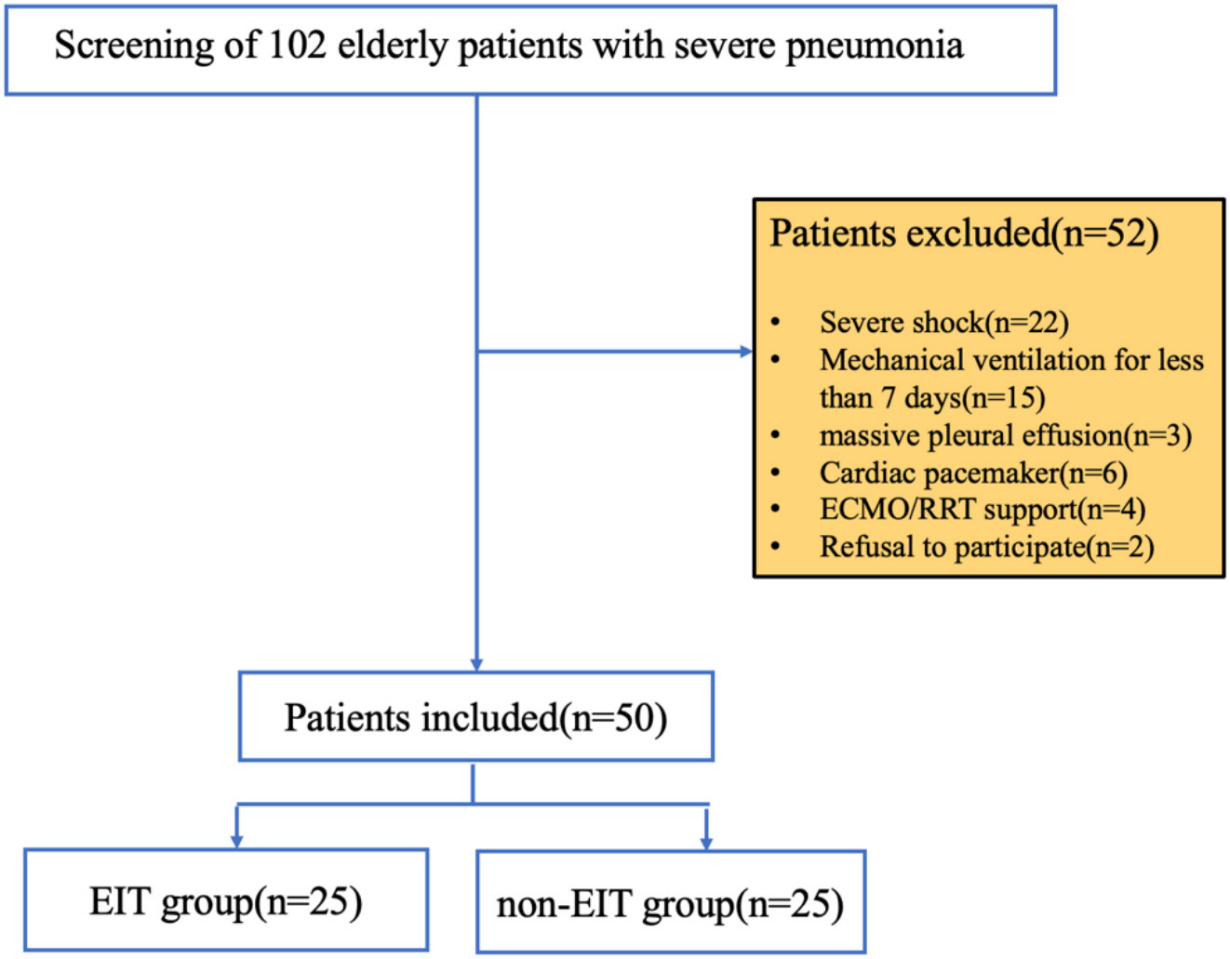
Study flowchart. ECMO, extracorporeal membrane oxygenation; EIT, electrical impedance tomography; RRT, renal replacement therapy.

A total of 102 elderly patients with severe pneumonia were screened for eligibility to participate in the study. Fifty patients who met the criteria for nativity were selected and randomly allocated into either the EIT group or the non‐EIT group using a random number table, with 25 participants in each group. The screening process is shown in Figure 1. There was no statistically significant difference between the baseline conditions of the two groups (*p* > 0.05; Table 1).

**TABLE 1:** Demographic and clinical characteristics of the EIT and non‐EIT groups.

Parameter	EIT group (*n* = 25)	Non‐EIT group (*n* = 25)	*p*
Demographics			
Age (years)	85.28 ± 9.23	83.20 ± 8.15	0.403
Weight (kg)	70.60 ± 6.97	67.88 ± 8.936	0.236
Gender (male, %)	19 (76.00)	16 (64.00)	0.355
APACHE II	22.20 ± 3.379	20.20 ± 5.895	0.1476
Laboratory results			
WBC (*10^9/L)	13.30 ± 4.590	14.70 ± 5.530	0.3363
PLT (*10^9/L)	169.3 ± 54.44	150.7 ± 45.09	0.1955
CRP (mg/L)	170.0 ± 56.53	155.3 ± 65.37	0.3970
PCT (mg/L)	1.733 ± 1.053	2.177 ± 1.563	0.2445
ALT (U/L)	26.24 ± 13.11	29.16 ± 14.21	0.4539
AST (U/L)	28.16 ± 12.80	30.72 ± 14.66	0.5138
TB (μmol/L)	14.04 ± 7.034	16.21 ± 10.29	0.3896
Scr (μmol/L)	85.28 ± 31.64	85.08 ± 45.32	0.9856
BUN (mmol/L)	9.720 ± 3.586	11.80 ± 7.745	0.2298
Comorbidities (*n*, %)			
Coronary artery disease	3 (12.00)	6 (24.00)	0.4635
Diabetes mellitus	8 (32.00)	6 (24.00)	0.7536
COPD	7 (28.00)	5 (20.00)	0.7416
Immuno suppressed state	9 (36.00)	5 (20.00)	0.3451
Malignant tumor	3 (12.00)	1 (4.00)	0.6092
CKD	2 (8.00)	8 (32.00)	0.0738
Mechanical ventilation parameters (*n*, %)			
Pressure‐support ventilation	10 (40.00)	16 (64.00)	0.1564
Assist‐control ventilation	15 (60.00)	9 (36.00)	0.1564
Concomitant medication(*n*, %)			
With Vancomycin	8 (32.00)	12 (48.00)	0.3868
With Linezolid	9 (36.00)	4 (16.00)	0.1963
With carbapenems	8 (32.00)	6 (24.00)	0.6489
With tigecycline	11 (44.00)	16 (64.00)	0.2563
With enzyme inhibitors	14 (56.00)	9 (36.00)	0.2563

Abbreviations: ALT, alanine aminotransferase; APACHE II, Acute Physiology and Chronic Health Evaluation II; AST, aspartate aminotransferase; BUN, blood urea nitrogen; CKD, chronic kidney disease; COPD, chronic obstructive pulmonary disease; CRP, C‐reactive protein C; EIT, electrical impedance tomography; PCT, procalcitonin; PLT, platelet; Scr, serum creatinine; TB, total bilirubin; WBC, white blood cell.

### 3.2 Comparison of Changes in Clinical Parameters Between Patients in the EIT and Non‐EIT Groups Over the Course of Treatment

**FIGURE 2:**
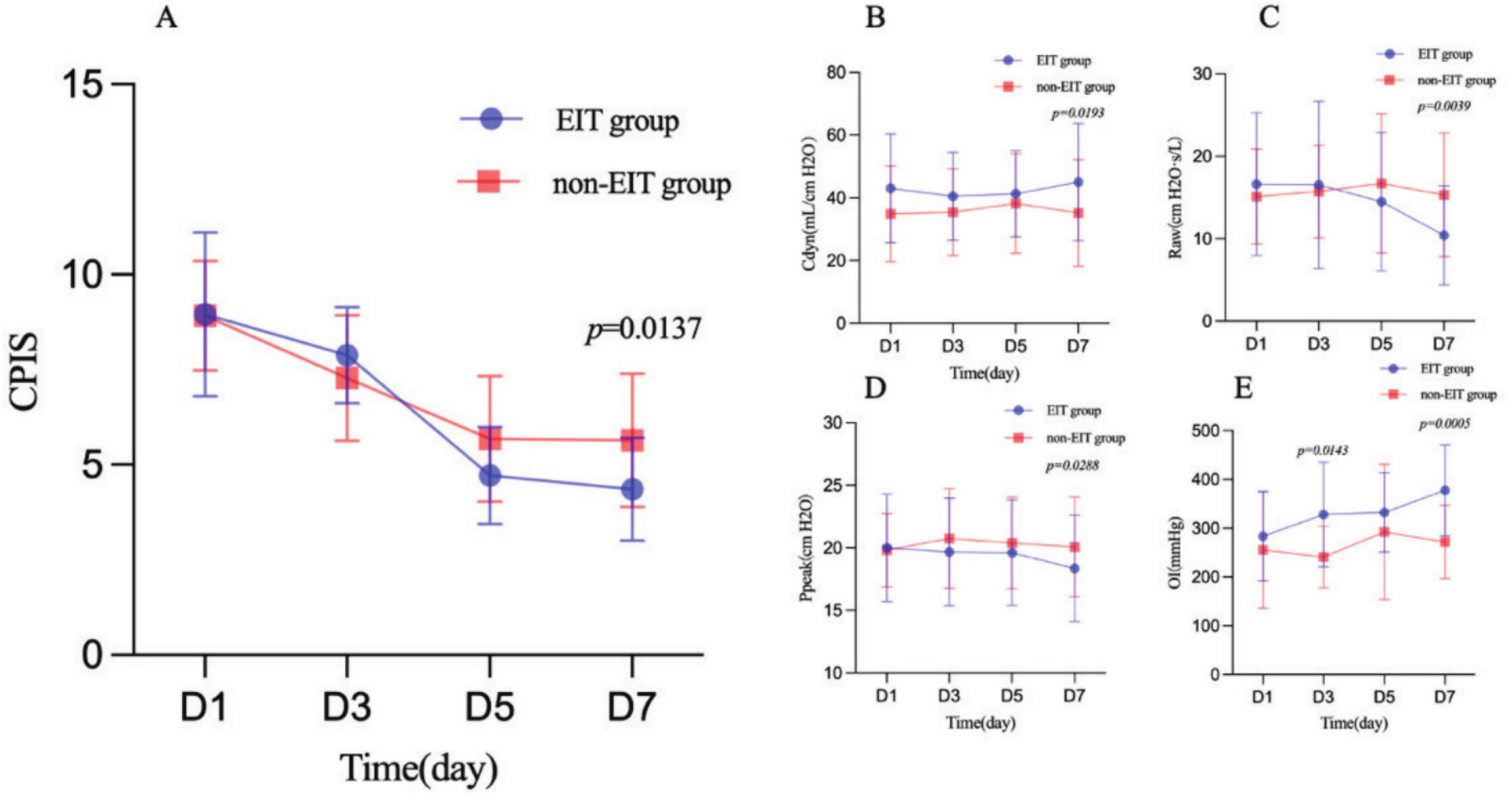
Comparison of clinical parameters between the two groups of patients at different time points. (A) Changes in CPIS over time, (B) changes in Cdyn over time, (C) changes in Raw over time, (D) changes in Ppeak over time, and (E) changes in OI over time. Cdyn, dynamic compliance; CPIS, Clinical Pulmonary Infection Score; EIT, electrical impedance tomography; OI, oxygenation index; Ppeak, peak airway pressure; Raw, airway resistance.

As shown in Figure 2, CPIS score in the EIT group was significantly lower compared with that in the non‐EIT group at treatment day D7 (EIT group vs. non‐EIT, *p* = 0.0137). Cdyn was higher in the EIT group compared with that in the non‐EIT group at treatment day D7 (EIT group vs. non‐EIT, *p* = 0.0193). Raw was lower in the EIT group compared with that in the non‐EIT group at treatment day D7 (EIT group vs. non‐EIT, *p* = 0.0039). Ppeak was lower in the EIT group compared with that in the non‐EIT group at treatment day D7 (EIT group vs. non‐EIT, *p* = 0.0288). OI was higher in the EIT group compared with that in the non‐EIT group at treatment days D5 and D7 (EIT group vs. non‐EIT, *p* = 0.0143, *p* = 0.0005).

### 3.3 Gas Distribution at Different Time Points in the EIT Group

**FIGURE 3:**
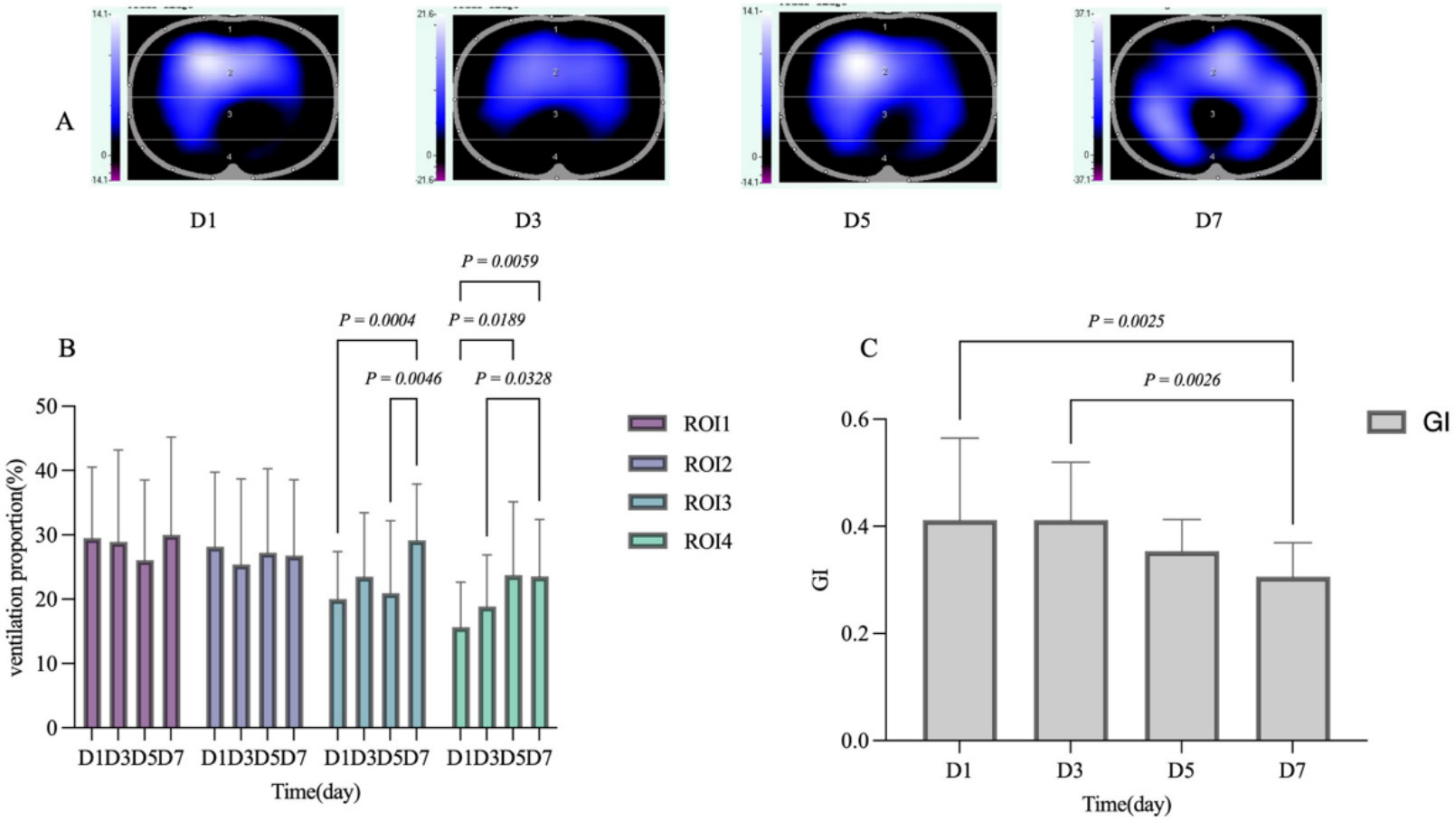
Gas distribution at different time points in the EIT group. (A) Pulmonary ventilation distribution map, (B) ventilation ratio at different time points, and (C) GI at different time points. GI, Global Inhomogeneity Index; ROI, region of interest.

As shown in Figure 3, ventilation in the ROI3 and ROI4 regions of the EIT group improved progressively over time (Figure 3A). In the ROI3 region, the proportion of D7 days ventilated gradually increased (D7 vs. D1, *p* = 0.0004; D7 vs. D5, *p* = 0.0046). In the ROI4 region, the proportion of D7 days ventilated gradually increased (D7 vs. D1, *p* = 0.0059; D7 vs. D3, *p* = 0.0328; D5 vs. D1, *p* = 0.0189).GI values were significantly lower at D7 (D7 vs. D1, *p* = 0.0025; D7 vs. D3, *p* = 0.0026).

### 3.4 Comparison of Patient Outcome Indicators Between the Two Groups

As shown in Table 2, the weaning success rate was significantly higher in the EIT group, with 22 successful cases (88.00%) compared with 14 (56.00%) in the non‐EIT group (*p* = 0.0255). After adjusting for potential confounders, the multivariate analysis revealed that the EIT group had a significantly lower risk of weaning failure compared with the non‐EIT group (Adjusted OR 0.1520, 95% CI 0.0264–0.6474, *p* = 0.0177). ICU length of stay and ICU mortality were not statistically significant in both groups.

**TABLE 2:** Clinical outcomes of the EIT and non‐EIT groups.

Outcome	Univariate analysis			Multivariate analysis	
	EIT group	Non‐EIT group	*p*	Adj.^a^ OR (95% CI)	*p*
ICU length of stay, days	13.08 ± 4.499	15.80 ± 5.212	0.0540		
ICU mortality, *n* (%)	3 (12)	6 (24)	0.4635		
Weaning success, *n* (%)	22 (88.00)	14 (56.00)	0.0255^a^	0.1520 (0.0264–0.6474)	0.0177^a^

Abbreviations: OR odds ratio; CI confidence interval; EIT Electrical Impedance Tomography.

^a^ Adjusted for age, sex, and APACHE II score.

## 4 Discussion

The primary results of this study indicate that elderly patients with severe pneumonia treated with EIT‐guided airway clearance experienced a significant reduction in the CPIS, along with improvements in ventilatory parameters and blood gas profiles throughout the treatment period. In terms of clinical outcomes, the EIT group demonstrated a superior rate of successful weaning from mechanical ventilation compared with the non‐EIT group.

Elderly patients with pneumonia often face a high risk of airway secretion retention due to decreased immunity, weakened cough reflex, and impaired mucociliary clearance, which can easily induce pulmonary atelectasis, hypoxemia, and secondary infections [16]. ACTs have an irreplaceable role in the treatment of elderly patients by facilitating secretion drainage through physical or physiological mechanisms [17]. By postural drainage combined with chest percussion vibration, gravity‐dependent principles are utilized to induce secretions from gravity‐dependent areas (e.g., ROI3–4) toward the central airway, reducing alveolar collapse and optimizing oxygenation [18]. ACBT enhances the efficiency of diaphragmatic activity through controlled deep breathing and coughing exercises, alleviating ineffective sputum coughing caused by respiratory muscle fatigue in elderly patients [19]. However, blind airway clearance maneuvers may lead to poor results and may even cause adverse reactions or complications. In our study, we aimed to develop personalized airway clearance strategies, guided by EIT, to improve the efficacy and safety of treatment.

Because EIT can dynamically display lung ventilation in real time and continuously at the bedside, EIT is commonly used at home and abroad to titrate PEEP and guide the ventilation strategy of mechanically ventilated patients with ARDS [8, 9, 20]. In this study, the effect of the ACT was monitored in time by observing EIT images in real time, so as to guide the patient to adopt the most effective position for clearance operation. The results of EIT monitoring in this study showed that the proportion of ROI3 and ROI4 gradually increased, and the GI index gradually decreased in the whole treatment process, and the results were statistically significant (*p* < 0.05). Increased ventilation in dorsal regions (ROI3–4) reflects the resolution of dorsal atelectasis and overall lung re‐expansion [21, 22]. The GI index is a deliberate index reflecting the uniformity of lung ventilation, and the decrease of its value indicates that the uniformity of the patient's ventilation has increased, indicating that the patient's collapsed or overinflated lung area has significantly improved during the treatment process [23, 24].

The CPIS score quantifies infection severity in critically ill patients, with higher scores denoting worse infection [25]. In this study, CPIS decreased in both groups after treatment, but the EIT group showed significantly lower CPIS than controls by Day 7. This indicates that the ventilation of the lungs can be observed more intuitively through the EIT‐guided ACT, and targeted treatment can be carried out in the diseased lung area. Effective removal and drainage of airway secretions from the deeper parts of the lungs help in the control of lung inflammation [26].

In current clinical practice, one of the routine methods for assessing the condition of mechanically ventilated patients and the effectiveness of treatment is to monitor the mechanical indexes of the ventilator, including parameters such as Cdyn, Ppeak, and Raw [27, 28]. The results of this study showed that the test group guided by the ACT combined with EIT showed significantly better improvement in all parameters than the control group. This finding suggests that EIT‐based real‐time monitoring during ACTs provides a quantitative assessment of lung status and gas distribution. Improved dorsal ventilation reduced intrapulmonary shunt by recruiting atelectatic alveoli, thereby enhancing ventilation‐perfusion matching and gas exchange [29, 30]. This result was validated by the improvement in the oxygenation index of blood gas results. This result emphasizes the importance of using an EIT‐guided personalized airway clearance strategy to optimize the patient's respiratory function during mechanical ventilation management.

As reported in previous studies, optimization of respiratory mechanics parameters and control of infections are key factors in weaning preparation [31, 32, 33]. The results of the present study further validate this view. In the present study, through the personalized implementation of ACTs under real‐time guidance of EIT, the EIT group had a higher success rate of weaning from mechanical ventilation than the non‐EIT group (EIT group vs. non‐EIT, 88% vs. 56%, *p* = 0.0255), whereas the respiratory mechanics and infection status improved in the EIT group. However, this study did not observe differences in hospitalization period and mortality between the two groups.

Our study also had some limitations. First, the sample size was small. We only enrolled patients from two centers; this may limit the generalizability of our findings. Second, the study focused only on short‐term clinical outcomes and lacked long‐term follow‐up data to assess the impact of EIT‐guided ACTs on patient prognosis.

## 5 Conclusion

In conclusion, this study demonstrates that EIT‐guided airway clearance significantly improves respiratory function and ventilator weaning success rates in elderly patients with severe pneumonia. The findings suggest that EIT provides a valuable tool for personalized airway clearance strategies, leading to optimized ventilation distribution, improved oxygenation, and reduced pulmonary inflammation. Furthermore, the enhanced weaning success rate observed in the EIT group highlights the potential of this technique to facilitate the recovery of critically ill patients.

## Author Contributions

J.P.Z., C.Y.Z., and W.M.C. contributed to data acquisition, analysis, and interpretation. C.Y.Z. and L.W. contributed to manuscript preparation. Y.Z., S.C.X., and W.H.H. performed the experiments. S.J.G. supervised the research and revised the manuscript. C.Y.Z. designed the research. All authors approved the final manuscript.

## Ethics Statement

This study was approved by the Ethics Committees of the Zhejiang Hospital(2020‐110K), and written informed consent was obtained from each patient's next of kin prior to participation.

## Consent

All authors have consented to the publication of the present manuscript.

## Conflicts of Interest

The authors declare no conflicts of interest.

## Data Availability

The data that support the findings of this study are available from the corresponding author upon reasonable request.
